# Perceptual Reorganization of Lexical Tones: Effects of Age and Experimental Procedure

**DOI:** 10.3389/fpsyg.2018.00477

**Published:** 2018-04-06

**Authors:** Antonia Götz, H. Henny Yeung, Anna Krasotkina, Gudrun Schwarzer, Barbara Höhle

**Affiliations:** ^1^Linguistics Department, University of Potsdam, Potsdam, Germany; ^2^Department of Linguistics, Simon Fraser University, Burnaby, BC, Canada; ^3^Developmental Psychology, Justus-Liebig University Gießen, Giessen, Germany

**Keywords:** perceptual reorganization, lexical tones, U-shaped curve, habituation, familiarization

## Abstract

Findings on the perceptual reorganization of lexical tones are mixed. Some studies report good tone discrimination abilities for all tested age groups, others report decreased or enhanced discrimination with increasing age, and still others report U-shaped developmental curves. Since prior studies have used a wide range of contrasts and experimental procedures, it is unclear how specific task requirements interact with discrimination abilities at different ages. In the present work, we tested German and Cantonese adults on their discrimination of Cantonese lexical tones, as well as German-learning infants between 6 and 18 months of age on their discrimination of two specific Cantonese tones using two different types of experimental procedures. The adult experiment showed that German native speakers can discriminate between lexical tones, but native Cantonese speakers show significantly better performance. The results from German-learning infants suggest that 6- and 18-month-olds discriminate tones, while 9-month-olds do not, supporting a U-shaped developmental curve. Furthermore, our results revealed an effect of methodology, with good discrimination performance at 6 months after habituation but not after familiarization. These results support three main conclusions. First, habituation can be a more sensitive procedure for measuring infants' discrimination than familiarization. Second, the previous finding of a U-shaped curve in the discrimination of lexical tones is further supported. Third, discrimination abilities at 18 months appear to reflect mature perceptual sensitivity to lexical tones, since German adults also discriminated the lexical tones with high accuracy.

## Introduction

During the first year of life, infants' perception abilities may change for stimuli that are not present or not relevant in their environment. For example, in the linguistic domain, perceptual changes have been detected in infants' sensitivity to native and non-native speech sounds. With increased experience with their native language, infants show an enhanced ability to distinguish between native speech sounds, whereas the initial sensitivity to non-native speech sounds decreases. This pattern of perceptual reorganization has been shown for consonants (Werker and Tees, 1984; Rivera-Gaxiola et al., 2005), vowels (Polka and Bohn, 1996, 2011; Tsuji and Cristia, 2014), lexical tones (Mattock and Burnham, 2006; Mattock et al., 2008; Yeung et al., 2013; Liu and Kager, 2014); (Singh and Fu, 2016), and word stress (Höhle et al., 2009; Skoruppa et al., 2009; Bijeljac-Babic et al., 2012).

However, research in recent years has converged on the idea that this picture is too simplistic. On the one hand, not all linguistically relevant sound contrasts are easily discriminable by young infants (Narayan et al., 2010; for a review, see Maurer and Werker, 2014). On the other hand, there are non-native sound contrasts that are discriminable by children beyond the typical ages of perceptual reorganization, and even by adults (for consonantal contrasts, see Best et al., 2001; for vocalic contrasts, see Mazuka et al., 2014). The present paper investigates the potential perceptual reorganization of lexical tones by infants learning non-tone languages. Previous research on lexical tone discrimination in infants is characterized by a rather complex pattern of findings: prior studies have found evidence for an increase, a decrease, and no-change in infants' and toddlers' ability to discriminate non-native tone contrasts across ages (for an overview, see Table 1). These divergent findings may be related to a number of dimensions on which these studies varied, including the tone contrasts used, the native language of the participants, and the experimental procedures. Our study focuses on the latter factor and compares the effects of familiarization vs. habituation in the initial exposure phase on German-learning infants' discrimination of a Cantonese tone contrast. In familiarization experiments infants are exposed to certain stimuli for a fixed time, thus the exposure is experimenter-controlled. In contrast, exposure in habituation is infant-controlled as the infant needs to reach a specific criterion (decrease in looking time) to proceed to the test phase. Thus, the latter type of pre-exposure may be more sensitive to the performance of individual infants.

**Table 1:** Summary of the previous results on infant lexical tone perception.

**No**	**Authors**	**Year**	**Age (months)**	**Native language**	**Contrast**	**Exposure phase**	**Results for non-tone group**
1	Chen and Kager	2016	4, 6, and 12	Dutch	Mandarin rising-low-dipping	Habituation	Perceptual enhancement
2	Chen at al.	2017	4 and 12	Dutch	Mandarin rising-low-dipping	Habituation	Perceptual enhancement
3	Liu and Kager	2014	5–6, 8–9, 11–12, 14–15, and 17–18	Dutch	Mandarin high-level-high-falling	Habituation	Discrimination across all ages; U-shaped curve (Discrimination 5–6 and 17–18 months)
4	Liu and Kager	2017	5–6, 8–9, 11–12, 14–15, and 17–18	Dutch bilinguals	Mandarin high-level-high-falling	Habituation	Discrimination across all ages; U-shaped curve (Discrimination 5–6 and > 11 months)
5	Mattock and Burnham	2006	6 and 9	English and Chinese	Thai rising-falling and rising-low	Conditioning	Perceptual Decline
6	Mattock et al.	2008	4, 6, and 9	English and French	Thai rising-low	Familiarization	Perceptual Decline
7	Ramachers et al.	2017	6, 9, and 12	Dutch and Limburgian	Limburgian falling-falling-rising	Habituation	Discrimination across all ages
8	Shi et al.	2017	4, 8, and11	French	Mandarin rising-low-dipping; high-level-falling	Habituation	Discrimination across all ages
9	Tsao	2017	6–8 and 10–12	English and Mandarin	Mandarin high-level-low-dipping	Conditioning	Perceptual Enhancement, but discrimination at both ages
10	Yeung et al.	2013	4 and 9	English, Cantonese, Mandarin	Cantonese high-rising-mid-level	Familiarization	Perceptual Decline

We will first review prior studies on infants' and adults' perception of lexical tones and then present three experimental studies. In the first study, Cantonese tone discrimination in adult native speakers of Cantonese was compared to that in adult native speakers of German. In the second study, the discrimination of the high-rising and the mid-level Cantonese tones was tested in German-learning infants between 6 and 18 months of age using a familiarization procedure. The third experiment investigated discrimination of the same tone contrast in 6- and 9-month-old German infants using a habituation procedure.

## Previous studies on infants' non-native lexical tone perception

A detailed review of infant tone perception can be found elsewhere (Singh and Fu, 2016). Here, we focus on studies that have investigated how infants learning a non-tonal language as their native language perceive different tones from various tone systems and we incorporate some more recent studies on infant tone perception. Furthermore, our review will also highlight details of prior experimental methods.

The first studies that tested perceptual reorganization of lexical tones provided evidence for a decline in tone discrimination by infants learning a non-tone language. Mattock and Burnham (2006) compared English and Chinese (Mandarin- or Cantonese-learning) infants at 6 and 9 months on their discrimination of Thai rising vs. falling as well as rising vs. low tones using the Conditioned Head-Turn (CHT) paradigm. Infants were first trained to perform a head-turn whenever an auditory background stimulus (a syllable carrying one tone) was replaced by the target stimulus (the segmentally same syllable with another tone). In the test phase—which was started after three consecutively correct head-turns in the training—the number of correct head-turns to a stimulus change was the dependent variable. Both 6- and 9-month-old Chinese-learning infants discriminated both tone contrasts, but English-learning infants showed a decrease in their discrimination from 6 to 9 months of age, with an overall higher performance for the rising-falling than for the rising-low contrast.

Mattock et al. (2008) extended this study to 4-month-old infants learning English or French, while continuing to test 6- and 9-month-olds acquiring these languages. They used a visual fixation paradigm (i.e., they measured infants' looking time at a central visual display during auditory stimulus presentation), where infants were initially exposed to a syllable representing either a low or a rising Thai tone for 30 s in a familiarization phase. In the test phase, two trial types were presented: four alternating trials that contained both the familiarized and the non-familiarized tone, and four non-alternating trials that only contained tokens of the familiarized tone. In this Stimulus Alternation Preference Procedure (SAPP), the 4- and 6-month-olds but not the 9-month-olds showed significantly longer looking times for the alternating trials compared to the non-alternating trials with no difference across the language groups.

Yeung et al. (2013) tested 4- and 9-month-olds learning Cantonese, Mandarin, and English on Cantonese tones that were similar to the Thai contrast (high-rising vs. mid-level tones) investigated by Mattock and colleagues. Using a modification of the SAPP, infants heard three trial types in the test phase: four alternating trials (familiarized and non-familiarized tone intermixed), two non-alternating trials only containing the familiarized tone, and two non-alternating trials only containing the non-familiarized tone. With this modification, discrimination and preference could be measured in the looking times obtained within the same experiment: that is, differences between the alternating and non-alternating trials would indicate discrimination while the direction of differences between the non-alternating trials would indicate preference. The English-learning infants showed a decline in the ability to discriminate these contrasts while this was not the case for the Mandarin or Cantonese infants. Moreover, infants learning one of the tonal languages showed an asymmetrical performance pattern with better discrimination when they were familiarized with the high-rising tone than with the mid-level tone.

While these studies showed a decline in discrimination ability for non-tone language learners, others have found enhanced perceptual abilities with increasing age (Chen and Kager, 2016; Chen et al., 2017; Tsao, 2017). Chen and Kager (2016) as well as Chen et al. (2017) tested Dutch-learning infants' discrimination of the Mandarin low-rising and low-dipping tones. Different from Mattock et al. (2008) and Yeung et al. (2013), who used familiarization in the initial exposure phase, infants were first habituated by repeatedly being exposed to one of the tones until their looking time had decreased for a predefined percentage. Then in the test phase, one trial of the habituated tone and one trial of the non-habituated tone were presented. The results from both studies suggest successful discrimination in 6- and 12-month-olds but not in 4-month-olds. The authors concluded from their results that, with increasing age, infants develop more fine-grained acoustic discrimination abilities for pitch information. Increasing perceptual sensitivity was also observed by Tsao (2017), who tested 6–8 and 10–12-month-old Mandarin- and English-learning infants using the CHT paradigm on the Mandarin high-level vs. low-dipping tones. Both language groups showed discrimination at both ages and their discrimination ability was enhanced with increasing age.

A third pattern found in the literature is that infants show no changes in their discrimination ability with increasing age (Liu and Kager, 2014, 2017; Ramachers et al., 2017; Shi et al., 2017; Tsao, 2017). Ramachers et al. (2017) tested Dutch and Limburgian^1^ 6-, 9-, and 12-month-old infants with Limburgian falling vs. falling-rising tones. After the infants were habituated with one tone, they were presented with trials that only contained the habituated tone (non-alternating) or with a mixture of the habituated and the non-habituated tones (alternating). Looking time to a central visual display was the dependent measure, and results showed that Dutch infants at all ages (with no previous exposure to this specific dialect) discriminated the Limburgian tone contrast. Ramachers et al. (2017) argue that Dutch intonation has pitch contours (H^*^L and H^*^LH%) that are acoustically comparable to the Limburgian tones (Gussenhoven, 2004), which may have led to a maintenance of discrimination. Shi et al. (2017) came to a similar result when testing French-learning 4-, 8-, and 11-month-old infants. They habituated the infants to one instance of two Mandarin tone contrasts: either one token from the perceptually close rising vs. low-dipping contrast or one from the perceptually more distinct high-level vs. falling contrast. Infants were then tested on their discrimination of the habituated and the non-habituated tones. The infants showed successful discrimination across all three age groups with slight indications of a decline only for the perceptually close contrast. They discuss their findings as an indication of the emerging impact of native phonology and of the acoustic salience of the tested contrast in the perception of the non-native tone patterns.

Finally, a fourth developmental pattern was observed by Liu and Kager (2014), who tested the discrimination of the Mandarin high-level vs. high-falling tonal contrast in Dutch infants between 5 and 18 months of age using the visual fixation paradigm implemented with a habituation procedure. Their study revealed perceptual sensitivity at all ages when using naturally recorded speech stimuli. However, they found a U-shaped developmental curve in a second experiment, in which synthesized stimuli with smaller acoustic differences of the same contrast were used. Specifically, Dutch-learning infants at 5–6 and 17–18 months of age discriminated the contrast in these materials, but not the intermediate age groups. This U-shaped development was also found in a group of bilingual infants learning Dutch and another non-tone language (Liu and Kager, 2017). In line with Shi et al. (2017), the authors interpreted the finding that Dutch-learning infants regain their ability to discriminate the tones as a result of their experience with the native (Dutch) intonation system and its modulation by the acoustic salience of the contrast. To our knowledge, the two studies by Liu and Kager (2014, 2017) are the only ones that have tested tone perception across a larger age range extending into the second year of life and that have found evidence for a U-shaped learning curve.

In sum, previous studies have shown that infants' non-native tone perception is probably influenced by a large number of factors, including age, task demands, the acoustic salience of the target tone contrast, and the prosodic systems of the native languages of the infant participants. Thus, developmental change in language acquisition and the experimental observation of this change seem to be dependent on a complex interaction of different factors. This links up with findings that show that older children and adult speakers of non-tone languages can also identify and discriminate lexical tones, even though their performance is typically below that of native speakers of the particular language (Burnham and Francis, 1997; Hallé et al., 2004; Francis et al., 2008; So and Best, 2010; Hay et al., 2015). The adult perception of L2 tones has been shown to be influenced by various factors, among others by the L1 lexical tone system (if the L1 is a tone language) or the use of pitch variation for post-lexical functions, (e.g., different intonation or phrasing patterns) in the native language (Wayland and Li, 2008; Caldwell-Harris et al., 2015), but also by specific task conditions (e.g., duration of the interstimulus interval, requirement to count backwards during the interstimulus interval) that can show differential effects on non-native and native speakers' performance (Lee et al., 1996). One explanation for good tone discrimination abilities in adult speakers of non-tonal languages is that hearers might adopt their knowledge about the native intonation system for identifying and discriminating lexical tones (Francis et al., 2008). For instance, Francis et al. (2008) found that English listeners were highly accurate in identifying the Cantonese high-rising tone, which the authors linked to the acoustic similarity of this Cantonese tone to the rising intonation pattern of questions in English. Another possibility derives from the acoustic salience of the tested contrast. Highly acoustically salient tone contrasts are easier to discriminate independent of the native language background (Hallé et al., 2004). Given these findings that tone discrimination in adult speakers of non-tonal languages is possible, but is modulated by several factors, adult speakers' performance also needs to be considered when studying perceptual reorganization of tone discrimination in early infancy.

## The current study

The above-reviewed research on infants' non-native tone perception reflects the influence of several factors on experimental outcomes: acoustic properties of the tones used in the experiments, characteristics of the prosodic systems of the native languages of the participants, and also aspects of the experimental procedures. The studies that have found a perceptual decline with increasing age have mainly used familiarization procedures (Mattock et al., 2008; Yeung et al., 2013), whereas all studies that have found patterns of (re-)increased or maintained sensitivity across age have used infant-controlled habituation or conditioning procedures (Liu and Kager, 2014, 2017; Hay et al., 2015; Chen and Kager, 2016; Chen et al., 2017; Ramachers et al., 2017; Shi et al., 2017; Tsao, 2017). This suggests that habituation may be the more robust procedure to reveal discrimination abilities in infants. In line with this consideration, a recent test–retest reliability study suggests that habituation results are more consistent and reveal larger effects at the group level than familiarization (Cristia et al., 2016). One reason for this could be that infants in a habituation procedure enter the test phase of the experiment on an individually controlled encoding status of the stimulus. The duration of the exposure during the habituation procedure is dependent on infants' response to the stimulus. In contrast, familiarization has a fixed duration that does not take into account individual differences in the speed of encoding the stimuli. According to the model by Hunter and Ames (1988), the degree of familiarity with the exposed stimulus (which depends on an interaction of stimulus complexity and the infants' age as an indicator of developmental level) determines whether an infant prefers the familiar or the novel stimulus in the test phase. Therefore, group results may reflect heterogeneous individual patterns of novelty or familiarity preferences, which may lead to null effects. This inconsistency in the direction of preferences is actually predicted after familiarization in some cases but is never predicted after habituation. Thus, the conflicting results on infants' tone perception obtained across different studies may at least partly be related to the use of different pre-exposure techniques.

The present study had two main objectives. First, we further investigated the U-shaped development found by Liu and Kager (2014) using another tone contrast and testing a population with a different native language than Dutch. To this end, discrimination of a Cantonese tone contrast was tested with German-learning infants between 6 and 18 months of age, as well as with a group of German and Cantonese adults. Second, we wanted to pursue the question of methodological impacts on the results in infant discrimination studies. For that reason, the effect of using a familiarization or a habituation technique on the discrimination performance of 6- and 9-month-olds was investigated by testing these two age groups with two different experimental procedures.

Before testing infants, we first asked whether the target tone contrast would be discriminated by adult speakers of German. We tested a group of German adults on their ability to discriminate Cantonese tone contrasts and compared the results to the performance of a group of adult native speakers of Cantonese. Our prediction was that German adults may be able to discriminate these tones in an AXB task but that Cantonese speakers should outperform the German speakers. An AXB task was chosen to reduce the effects of memory load. Different tokens of syllables from the same tonal category were used to force listeners to discriminate categorically rather than acoustically.

## Experiment 1: adults' discrimination of cantonese lexical tones

### Methods

#### Participants

Ten native Cantonese speakers (19–31 years, 5 female) and 14 native German speakers (22–31 years, 8 female) participated in this study. None of the native German speakers had any language competence in Cantonese or another tone language. Although all participants reported L2 proficiency in English, they considered themselves to be monolingual. All participants reported normal hearing abilities. The study was approved by the Ethics Committee of the University of Potsdam. Written informed consent in accordance with the Declaration of Helsinki was obtained from all participants.

#### Stimuli

The stimuli for the adult experiment comprised five different Cantonese lexical tones: high-rising (Tone 25), mid-level (Tone 33), low-falling (Tone 21), low-rising (Tone 23), and low-level (Tone 22). Although our experiments with the German infants (see below) were restricted to testing the discrimination of only Tone 33 and Tone 25^2^, we examined more tone contrasts in the adult experiment. This was done in order to minimize any effects of only presenting two tones repeatedly, which may draw the participants' attention to their specific acoustic differences and thus foster enhancement of discrimination during the experiment. A second reason for including multiple tones was to generate a broader picture of German adults' processing of lexical tones.

**Figure 1:**
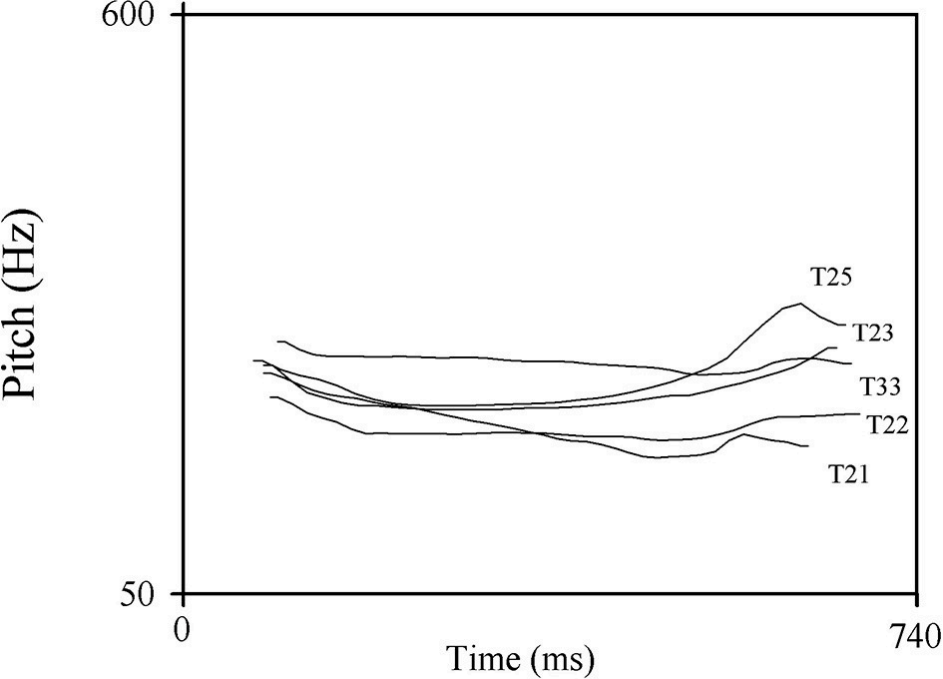
An example of the F0 contours of the syllable /jin/ of the five different tested Cantonese tones.

A female native speaker of Cantonese produced 40 segmentally different CV and CVC syllables in each of these five tones leading to 200 different syllables overall (e.g., the syllables/jin/and/se/, each produced with five different tones). Half of the stimuli were CV and the other half CVC syllables. All syllables had a legal German phonotactic structure and were meaningful Cantonese words. To create acoustic variability the speaker produced each stimulus four times. An acoustic analysis of the pitch patterns of the stimuli was conducted using PRAAT (see Table 2; Boersma and Weenink, 2016). Pitch contours were measured by sampling at three different time points within the vowel: at initial, middle (at 50%), and final position. Figure 1 illustrates an example of the five different pitch contours of the syllable/jin/. The pitch contour of level tones showed no change across the syllable (Tone 22, Tone 33), whereas for contour tones a pitch rise (Tone 23, Tone 25) or fall (Tone 21) occurred at the end of the syllable. For the experiment, all stimuli were normalized in intensity.

**Table 2:** Results from the acoustic analysis of the different Cantonese lexical tones.

**Tone**	**F0 initial in Hz**	**F0 middle in Hz**	**F0 final in Hz**
21	183 (20)	168 (17)	162 (20)
23	176 (16)	187 (17)	214 (16)
25	183 (12)	193 (14)	229 (12)
22	198 (16)	191 (16)	193 (16)
33	211 (17)	206 (18)	207 (17)

*All values are f0 means, Standard Deviations are given in parentheses. The analysis was done at three different positions: at the initial, middle and final position of the pitch contour*.

#### Procedure

Both Cantonese and German adults performed an AXB discrimination task. In this task, participants needed to discriminate between ten different tone pairs. The five tone types were combined with each other, such that Stimulus A and B of a trial were always segmentally identical syllables but belonged to different tone categories; X also had the same segmental structure and belonged either to the same tone category as A or as B. An AXB task was chosen to reduce the effects of memory load compared to an ABX task. The X in an AXB task is equally distant from A to B, which prevents a mapping bias to the B stimulus (Best et al., 2001; Hallé et al., 2004; Strange and Shafer, 2008). Within a trial, different tokens of the syllables from the same tonal category were used to force listeners to discriminate categorically rather than acoustically (Best et al., 1988; Polka, 1991, 1992), thereby increasing the likelihood of finding language-specific effects.

Four different trial types with the four possible orders of the stimuli were presented: AAB, ABB, BAA, and BBA. Each participant heard each of the 40 types of syllables combined with only one tone contrast. The pairing was randomized and counterbalanced across the participants (e.g., one participant heard the contrast Tone 25–Tone 33 on the syllable/se/, while another participant heard the contrast Tone 22–Tone 33 on the same syllable). Therefore, every participant heard each of the 40 syllables during the experiment but the tone contrast that was instantiated on these syllables varied across the participants. Each tone contrast occurred with four different syllables for each participant. During the experiment, each syllable-tone pairing was presented four times, once in each trial type. This resulted in an overall number of 160 trials for each participant (4 syllables × 10 tone contrasts × 4 trial orders). These trials were divided into four blocks of 40 trials, in order to allow pauses in between. Each block only contained one of the trial types for a syllable-tone pair. The trials within a block were presented in a pseudo-randomized order with the same tone contrast never repeating twice in row. The stimuli within trials were separated by an interstimulus interval of 1,000 ms; the intertrial interval was 3,000 ms. An interstimulus interval of 1,000 ms was chosen because previous studies have shown that language-specific effects are more clearly revealed with long interstimulus intervals (Werker and Logan, 1985). The maximum response time for the participants was 2,500 ms, measured from the offset of the last syllable. The pause between blocks was controlled by the participant, and the experiment continued when the participant pressed a button. In total, the experiment lasted around 20 min.

Participants were instructed to decide whether the second syllable was more similar to the first or to the third syllable, otherwise they were not instructed to attend to any specific part of the syllables. The experiment and the participants' responses on a keyboard were controlled with OpenSesame (Mathôt et al., 2012) and run on a laptop. All trials were presented over headphones in a silent room.

### Results

**Figure 2:**
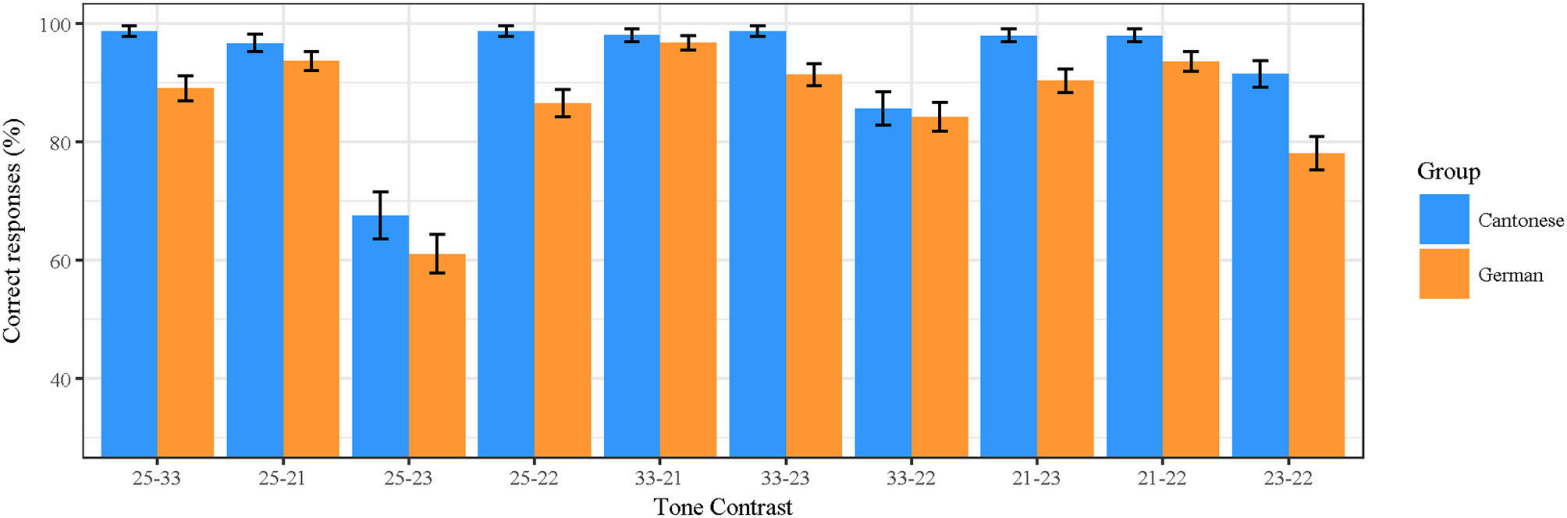
Results from the AXB discrimination task, separated by group and tone contrast.

Figure 2 summarizes the percentages of correct responses given for all contrasts by both language groups. Statistical analyses were run on the number of correct responses as the dependent variable. The performance of both language groups was significantly higher than predicted by chance for all tone contrasts (one sample *t*-test against chance level, all *p's* < 0.001). This was also true for the relevant tone contrast for the infant study (Tone 33–Tone 25). Most importantly, a one sample *t*-test against chance revealed above chance performance in German adults (*t* = 18.55, *p* < 0.001) for this contrast.

As a next step, we compared different models that were computed with the function glmer from the lme4 package (Bates et al., 2015) in R (R Core Team, 2017). Models and their results were obtained by the anova function. The best fitting model [lowest Akaike Information Criterion (AIC, Akaike, 1998) and significant difference in the Chi-square test] included item and subject as random factors and interaction of language group (Cantonese and German) and tone contrast (the 10 different tone contrasts) as fixed factors; see Table 3. Additionally, we asked for musical experience. Participants were asked whether they had learned to play an instrument and if yes, how long they do or did play it. Model comparison revealed that musical experience (years playing an instrument) did not modulate the outcome of our data. Compared to the model including the interaction of Tone Contrast and Language group, the model including musical experience has higher AIC (2183.4 compared to 2175.6) and no significantly better fit with Chi-square test results (*p* = 0.19).

**Table 3:** Results from the model comparison of the adult perception experiment.

**Model**	**Df**	**AIC**	**BIC**	**logLik**	**Deviance**	**Chisq Chi**	**Df**	**Pr(>Chisq)**
~Tone contrast + (1|subject)	11	2,187.6	2,255.6	−1082.6	2,165.2			
~Tone contrast + (1|subject) + (1|item)	12	2,160.8	2,235.5	−1068.4	2,136.8	28.397	1	<0.001^***^
~Tone contrast ^*^ Group + (1| subject) + (1|item)	22	2,275.6	2,275.6	−1047.3	2,094.7	42.114	10	<0.001^***^

Results from the model comparison of the adult perception experiment. The comparison is organized hierarchically. The first model was compared to the second model – which fit better to the data. The second model was then compared to the third and so forth. The comparison revealed best fit for the model which includes the interaction of tone contrast and group as fixed effect and subject and item as random effects (

*** *indicates p < 0.001)*.

In general, our results reveal good performance in both groups, but show that German native listeners performed less accurately than the native Cantonese listeners (86.5 vs. 93.4%, respectively). The statistical analysis showed that the overall performance differed significantly between the two language groups (β = −2.253, *SE* = 0.758, *z* = −2.973, *p* < 0.01). However, this group difference was not significant across all contrasts as indicated by the interaction of tone contrast with group. Cantonese listeners best discriminated high-rising (25) vs. mid-level (33), high-rising (25) vs. low-level (22), and mid-level (33) vs. low-rising (23), each at a level of 98.7%. German adults performed best on the discrimination of mid-level (33) vs. low-falling (21). For both groups, the contrast high-rising (25) vs. low-rising (23) was the most difficult contrast.

With respect to the infant experiments, we were especially interested in how native and non-native adults perceive the difference between high-rising and mid-level tones. Our results revealed that the Cantonese adults discriminated Tone 25 vs. Tone 33 significantly better than the German listeners (β = −2.503, *SE* = 0.871, *z* = −2.874, *p* < 0.01). Furthermore, native listeners discriminated Tone 25 vs. Tone 22 (β = −2.567, *SE* = 0.786, *z* = −3.265, *p* < 0.01), Tone 33 vs. Tone 23 (β = −2.047, *SE* = 0.850, *z* = −2.409, *p* < 0.01), Tone 21 vs. Tone 23 (β = −1.818, *SE* = 0.713, *z* = −2.549, *p* < 0.05), and Tone 23 vs. Tone 22 (β = −1.127, *SE* = 0.336, *z* = −3.358, *p* < 0.001) significantly better than the non-native German listeners. The discrimination for the other tone contrasts was not significantly different between the Cantonese and the German listeners.

### Discussion

The first experiment tested the discrimination of Cantonese lexical tones by adult German listeners without knowledge of Cantonese and by native speakers of Cantonese. Three main findings were obtained: First, German native speakers were able to distinguish between different lexical tones. Second, native Cantonese speakers outperformed German listeners in their overall discrimination abilities. Third, there was variation in German listeners' discrimination performance depending on the specific contrast: while the discrimination reached native-like levels for some contrasts, performance was below that of native speakers for other contrasts. This is in line with other discrimination studies that have shown good discrimination by non-native listeners, but an overall better performance by native listeners (Lee et al., 1996; Burnham and Francis, 1997; Cutler and Chen, 1997; Francis et al., 2008).

However, the picture becomes less clear when comparing performances of each tone contrast separately. Some lexical tones (high-rising vs. mid-level, high-rising vs. low-level, low-rising vs. mid-level, low-rising vs. low-level, and low-rising vs. falling) are harder to discriminate for German than for Cantonese native speakers. However, there are also contrasts for which both language groups show comparable levels of high performance (high-rising vs. low-falling, mid-level vs. low-falling, and low-level vs. falling). Further, there are two contrasts for which both language groups show comparably lower performance (high-rising vs. low-rising, mid-level vs. low-level). It is striking that the pairs that are highly discriminable by both groups contain one level and one contour tone or two contour tones with frequency changes in opposite directions, while the tone pairs that are harder to discriminate are both level tones or show the same direction of frequency change. This pattern suggests that for non-native as well as for native tone discrimination, acoustic properties and the acoustic distance of the specific tone contrast are relevant for their discriminability. In addition, it is possible that German listeners assimilate some of the tones to their native intonation system. This would then support a language-specific account of adult tone perception. It is noteworthy that all contrasts that are highly discriminable for the German listeners contain the falling Tone 21. The good discrimination seen here might stem from familiarity with the German intonation system, which uses falling contours for neutral statements (Grice and Baumann, 2002). That is, similar to what Francis et al. (2008) have proposed for English listeners, German native speakers might use their knowledge of the native intonation system to discriminate non-native lexical tones.

To summarize, our findings from the first experiment revealed that German native speakers discriminate Cantonese lexical tones highly accurately, but native listeners perform significantly better. The overall good discrimination performance for German listeners could be explained by acoustic salience and/or assimilation to the native prosody. Our results thus showed that native and nonnative adults' performance may differ depending on the specific contrast. Discrimination abilities in adults should therefore be considered before testing potential changes in infants' non-native sound discrimination. Overall, the most important finding from our first experiment is that German adults can discriminate the tone contrast that was used in our infant studies (Tone 33 vs. Tone 25), but that their performance was below that of native speakers of Cantonese. The finding that German adults can hear the difference between these tones increases the likelihood of observing a U-shaped developmental pattern, or perceptual enhancement with increasing age. But the finding that native Cantonese listeners show higher achievements in discriminating these two tones suggest that their discrimination is not only due to a large acoustic distance, but is also affected by the native language of the listener.

## Experiment 2: testing 6-, 9-, and 18-month-olds using a familiarization procedure

Here we contribute new data to the infant tone perception literature by testing German infants' perception of the Cantonese Tone 33 vs. Tone 25 contrast that had previously been used in a study with English-learning infants by Yeung et al. (2013). Similar to Liu and Kager (2014), we included a wider age range than Yeung et al. had done in order to test for evidence of a U-shaped developmental curve in German 6-, 9-, and 18-month-olds. Following the Yeung et al. study, we used a procedure involving familiarization, but the discrimination abilities during the test phase were assessed with the head-turn preference procedure.

### Methods

#### Participants

In total, 88 monolingual German-learning infants participated in this experiment: 30 6-month-olds (*M*_*age*_ = 182 days; *range* = 168–194 days; 14 girls), 30 9-month-olds (*M*_*age*_ = 275 days; *range* = 258–289; 18 girls), and 28 18-month-olds (*M*_*age*_ = 540 days; *range* = 526–556 days; 13 girls). An additional 16 infants were tested, but excluded from the analysis for the following reasons: crying (*n* = 8), fussiness (*n* = 5), technical error (*n* = 1), and pre-term (*n* = 2). Another two infants were excluded because at least one of the main caretakers grew up in an area in which the local German dialect uses word-level pitch contrasts (Werth, 2011). The remaining infants were all born full-term. According to parental report, infants did not suffer from repeated or acute ear infections, and there were no indications of atypical development or any experience with a tone language. This study was carried out in accordance with the recommendations of the Ethics Committees of the University of Potsdam with written informed consent given by the parents in accordance with the Declaration of Helsinki.

#### Stimuli

For this study, we used the stimuli from Yeung et al. (2013): Cantonese CV syllables (/tɕ^h^i/) with either a high-rising (Tone 25) or mid-level (Tone 33) tone. In total, there were four different tokens of each tone. For detailed acoustic properties of the syllables, see Yeung et al. (2013).

The familiarization phase included only tokens of either Tone 25 or Tone 33. During the test phase, single syllables were concatenated into two different types of sequences: non-alternating (tokens from one tone category) and alternating sequences (tokens from both tone categories). In total, the test phase contained eight trials: four non-alternating and four alternating trials. Two of the non-alternating trials included only tokens of Tone 25 and the other two only of Tone 33. In the alternating trials, tone types were intermixed: the first four tokens at the beginning of the trial alternated between the two tones, the following ones were in a random order. The tokens were separated by an interstimulus interval of 1 s. Half of the alternating trials started with Tone 25, the other half with Tone 33, and they contained the same number of both tone types. During the familiarization phase, the maximal trial length was 15 s and during the test phase it was 30 s.

#### Procedure

The experiment was run with the head-turn preference procedure (Hirsh-Pasek et al., 1987; Jusczyk and Aslin, 1995), which differed from Yeung et al.'s use of visual fixation, but still measured auditory preference by recording the duration of attention to a visual stimulus while being presented to an acoustic stimulus. Infants sat on their caretakers' lap in a booth and first fixated on a flashing green lamp in front of them. Next, the experimenter—who sat in a second room and monitored the infants' gaze via a camera mounted above the green light—started the experimental trial by pressing a button. Then, one of the red lights mounted on the left or the right side inside the booth began to flash. As soon as the infant fixated the now blinking red light, the experimenter started the acoustic stimulus. The trial ended when the infant either looked away for more than 2 s, or when the end of the acoustic stimulus was reached. To start the next trial, the experimenter pressed a button and the green light in front of the infant again began to flash. Infants' looking duration (listening time) was coded online by the experimenter via a button box connected to a computer.

The experiment consisted of a familiarization and a test phase. During the familiarization phase, infants were presented with only one of the tones (either Tone 25 or Tone 33, counterbalanced across participants) until they had accumulated 30 s of listening time. A maximal trial length of 15 s assured that the infant looked at least once to both sides of the sound source during the familiarization. The test phase followed immediately after the familiarization phase and consisted of eight trials: two non-alternating trials of Tone 25, two non-alternating trials of Tone 33, and four alternating trials. These eight test trials were the same for all infants. During the test phase, the presentation order of alternating and non-alternating trials was pseudo-randomized; two alternating or non-alternating trials never followed each other directly (i.e., N-A-N-A-N-A-N-A or A-N-A-N-A-N-A-N). The test phase was additionally divided into two blocks: in each block, each trial type (alternating, non-alternating Tone 25, non-alternating Tone 33) was presented at least once. The presentation order of alternating, non-alternating Tone 25, and non-alternating Tone 33 was counterbalanced across infants, so that each of the trial types was presented in every position during the test phase. To check the reliability of the online measures of listening time (which was automatically calculated based on the experimenter's button pressing), 50% of the videos (randomly selected) obtained during the experimental session were re-coded by a second experienced coder using specialized software ELAN (Wittenburg et al., 2006). The inter-coder reliability was Pearson's *r* = 0.99, *p* < 0.001.

### Results

**Figure 3:**
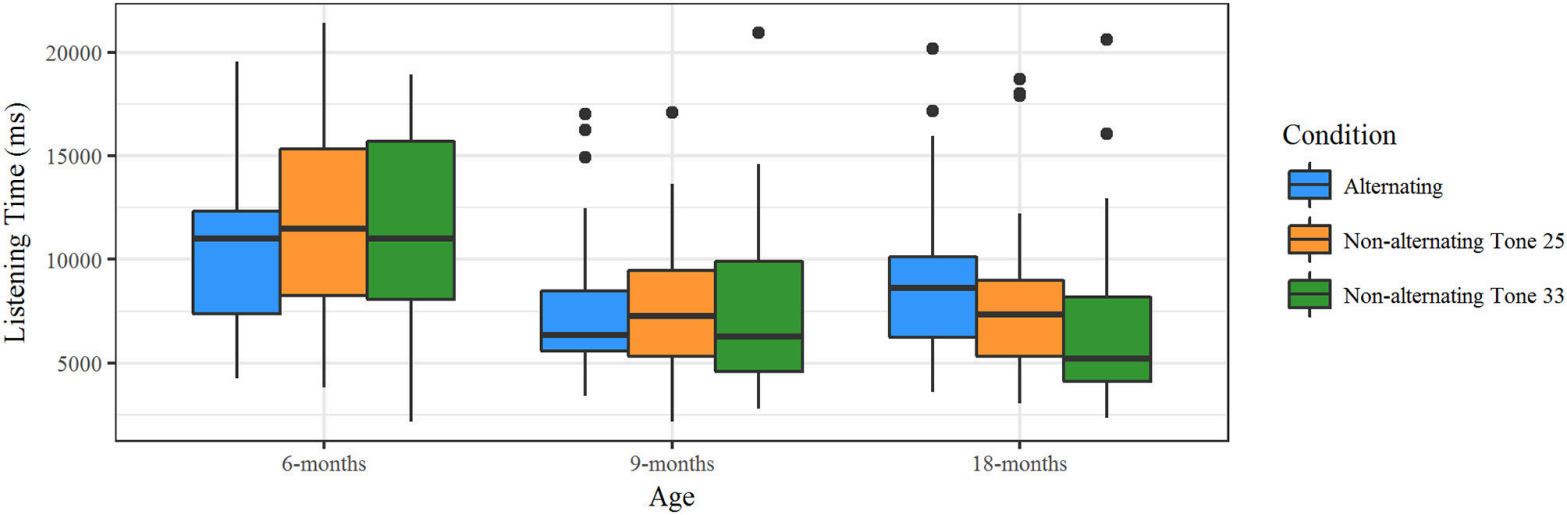
Results from the familiarization experiment divided by age group. Mean listening times for the alternating trials were only significantly longer at 18 months, indicating that only the 18-month-olds discriminated the lexical tones.

The averaged listening times for each trial type were entered as dependent variable into the statistical analysis. The mean listening times separated by age group and condition are displayed in Figure 3. For the statistical analysis, listening times were logarithmically transformed in order to create a normal distribution of the residuals. Data were analyzed with R (R Core Team, 2017) and linear mixed models with the lmer function from the package lme4 (Bates et al., 2015). Model comparison revealed that the model including the interaction of Condition (alternating, non-alternating Tone 25, and non-alternating Tone 33) × Age Group (6-, 9-, and 18-months) as fixed effect and trial number and subject as random factors fit best to our data (Table 4). This indicates that the listening times are differently affected by the conditions and the age. Furthermore, the comparison revealed that the tone used for the familiarization did not modulate the results, as including this factor did not improve the model fit (indicated by higher AIC and no significant difference in the Chi-square test).

**Table 4:** Results from the model comparison of the familiarization paradigm.

**Model**	**Df**	**AIC**	**BIC**	**logLik**	**Deviance**	**Chisq**	**Chi Df**	**Pr(>Chisq)**
~Condition + (1|subject)	5	1,515.6	1,538.3	−752.78	1,505.6			
~Condition^*^Age + (1|subject)	11	1,502.5	1,552.7	−740.26	1,480.5	25.04	6	<0.001^***^
~Condition + (1|subject) + (1|trial_number)	12	1,457.6	1,512.2	−716.78	1,433.6	47.00	1	<0.001^***^
~Condition^*^Age^*^ Familiarization + (1|subject) + (1|trial_number)	21	1,469.5	1,565.1	−713.72	1,427.5	60.11	9	0.729

Results from the model comparison of the familiarization paradigm. The comparison is organized hierarchically. The first model was compared to the second model – which fit better to the data. The second model was then compared to the third and so forth. Trial number refers to each individual trial, familiarization refers to the type of familiarization tone. Results from the Chi-square test and AIC score revealed best model fit for the model which includes the interaction of Age and Condition as fixed effect and subject and trial number as random effects (

*** *indicates p < 0.001)*.

As the model showed a significant interaction of Age Group × Condition, we calculated separate models for each age group. Detailed statistical information for all age groups is provided in Table 5. These models also included subject and trial number as random factors and Condition as fixed effect. Familiarization was not included as a fixed effect, as the previous general model did not show an effect for the familiarization tone.

**Table 5:** Detailed results of the statistical analysis of the familiarization experiment for each age group.

**Fixed effects**	**Estimate β (SE)**	**df**	***t*-value**	**Pr(>|t|)**
**6-MONTH-OLDS**				
(Intercept) Alternating	3.923 (0.062)	17.1	63.809	<0.001
Non-alternating tone 25	0.066 (0.040)	203.1	1.677	0.095
Non-alternating tone 33	0.041 (0.040)	203.1	1.036	0.302
**9-MONTH-OLDS**				
(Intercept) Alternating	3.788 (0.037)	21.14	102.033	<0.001
Non-alternating tone 25	−0.004 (0.041)	202.52	−0.099	0.921
Non-alternating tone 33	−0.038 (0.041)	202.52	−0.926	0.356
**18-MONTH-OLDS**				
(Intercept) Alternating	3.834 (0.043)	21.43	89.972	<0.001
Non-alternating tone 25	−0.031 (0.049)	189.65	−0.616	0.539
Non-alternating tone 33	−0.104 (0.049)	186.65	−2.097	0.037^*^

Detailed results of the statistical analysis of the familiarization experiment for each age group. The estimates represent the log-transformed listening times. The results indicate that only the 18-month-olds discriminate the contrast by longer listening times to the alternating trials, but not the 6- and 9-month-old infants. All models included Condition as fixed effect and subject and trial number as random effects as revealed as the best fit by the overall model comparison (

* *indicates p < 0.05)*.

For the 6-month-olds, the listening times for the alternating trials (*M* = 10.6 s, *SD* = 7.9 s) did not differ significantly from the listening times for the non-alternating Tone 25 trials (*M* = 11.9 s, *SD* = 7.6 s) nor from those for the non-alternating Tone 33 trials (*M* = 11.3 s, *SD* = 7.9 s). The effect sizes (Cohen's *d*) for alternating vs. non-alternating Tone 25 were *d* = −0.249, and for alternating vs. non-alternating Tone 33 *d* = −0.108.

The 9-month-olds also did not show significant differences in their listening times for the alternating trials (*M* = 7.63 s, *SD* = 5.17 s) compared to the non-alternating Tone 25 (*M* = 7.55 s, *SD* = 4.89 s) or the non-alternating Tone 33 (*M* = 7.53 s, *SD* = 5.93 s) trials. The effect sizes (Cohen's *d*) for alternating vs. non-alternating Tone 25 were *d* = −0.009, and for alternating vs. non-alternating Tone 33 *d* = 0.132.

However, the 18-month-olds showed significantly longer listening times for the alternating trials (*M* = 9.07 s, *SD* = 6.87 s) than for the non-alternating Tone 33 trials (*M* = 6.89 s, *SD* = 5.47 s). The difference between alternating and non-alternating Tone 25 trials (*M* = 8.15 s, *SD* = 5.90 s) was not significant. The effect sizes (Cohen's *d*) for alternating vs. non-alternating Tone 25 trials were *d* = 0.087, and for alternating vs. non-alternating Tone 33 trials *d* = 0.323.

### Discussion

The results from this experiment did not provide evidence that 6- and 9-month-old German-learning infants discriminate the Cantonese Tone 25–Tone 33 contrast. Only the 18-month-olds showed discrimination abilities for this contrast. However, discrimination showed up only in the comparison of the listening times to alternating sequences and non-alternating sequences containing Tone 33. No evidence of discrimination occurred between alternating sequences and non-alternating sequences that only contained Tone 25. This indicates some kind of asymmetry in the perception of these tones by German 18-month-olds.

Taken together, these results are only partly congruent with our prediction of perceptual reorganization and a U-shaped learning curve in tone perception. On the one hand, the differences in the results between the 9- and the 18-month-olds are in line with the observations by Liu and Kager (2014), who report an increase in the discrimination of Mandarin tone contrasts by Dutch-learning infants across these ages. Furthermore, our finding that 18-month-olds discriminate the tones is in line with our findings from Experiment 1 since German adults can also discriminate this contrast. However, what is missing is evidence of a decline in perceptual sensitivity between 6 and 9 months of age, as neither the 6- nor the 9-month-olds gave any indication of discriminating the contrast. So far, our result pattern for German-learning children is mostly compatible with the hypothesis of an age-related enhancement in tone perception, which is consistent with the findings of previous studies with Dutch-learning (Chen and Kager, 2016; Chen et al., 2017) or English-learning (Tsao, 2017) infants. Given the fact that German 7- to 8-month-old infants have been shown to be sensitive to pitch variations (Wellmann et al., 2012; Abboub et al., 2016), the assumption that even 9-month-olds may not yet be able to discriminate the tone contrasts based on pitch information is not likely. However, it might be that infants at this age focus on sound contrasts that mark lexical distinctions in their native language. Since this is not the case for pitch differences on the syllabic level, 9-month-olds might ignore these pitch differences.

There may be at least two other potential explanations for our failure to find indications of a decline in discrimination in the two younger age groups that we tested. The first one is that perceptual reorganization for these tone contrasts has set in before 6-months of age. Remember that Yeung et al. (2013) tested 4- and 9-month-old but not 6-month-old English-learning infants with the same tone contrasts as were used with the German infants. They found discrimination in 4-month-olds but not in the 9-month-olds. Comparing the English-, Mandarin- or Cantonese-learning 4-month-olds in that study revealed that all language groups discriminated between the tones, but that the preference patterns for the different stimulus types were not the same across the groups. This suggests language-specific influences on tone perception already at this early age, leaving open the possibility that we would have found evidence for perceptual reorganization in German infants younger than 6 months. Nevertheless, a number of other studies using different stimuli and testing infants exposed to different languages found non-native tone discrimination in 6-month-olds (Mattock and Burnham, 2006; Mattock et al., 2008). This suggests that the perceptual decline for lexical tone contrasts is not necessarily completed by the age of 6 months.

A second explanation for our failure to find evidence for changes in the younger infants' tone perception is methodological in nature: the method used in our experiment may not have been suitable to demonstrate infants' ability to discriminate the tones. As argued above, the effect of familiarization may be modulated by characteristics of the stimuli and the participants, making this type of pre-exposure not optimally suitable to uncovering discrimination abilities for all types of stimuli at all ages. Hence, our third experiment reinvestigated 6- and 9-month-olds' discrimination of the same contrasts as in the previous experiment but using a habituation procedure during the exposure phase.

Before we come to the third experiment, the results of the 18-month-olds deserve some consideration. As stated above, their listening times were longer for the alternating trials compared to the Tone 33 non-alternating trials, but not compared to the Tone 25 non-alternating trials. This pattern seems to be caused by enhanced listening times for the non-alternating Tone 25 sequences (compared to the non-alternating Tone 33 sequences). Listening times reflect specific preferences that infants have for stimuli that are presented during the experiment, and such preferences can emerge in the course of the experiment (when a familiarization phase is included) or can also be caused by some inherent properties of the stimuli (e.g., acoustic saliency, familiarity, etc.). Our results suggest that for German-learning infants, high-rising tones attract more attention compared to mid-level tones. Interestingly, Yeung et al. (2013) also found that the Mandarin-learning (but not the Cantonese-learning) infants showed longer listening times to Tone 25 compared to Tone 33. In contrast, the English-learning 4-month-olds showed a preference for listening to Tone 33 compared to Tone 25. The authors suggested that these differences in preference speak against an acoustic explanation that applies across languages, but rather suggests a language-specific preference for a certain tone type. A similar explanation may hold for the results of the German 18-month-olds. Their greater attention to Tone 25 than to Tone 33 indicates that they prefer pitch contours over level tones, which may be driven by the function that pitch contours have in German. In intonation languages like German, rising pitch contours often occur at the end of clauses, where a pragmatic function is to mark the utterance as a question or to indicate that the sentence is not yet finished (Grice and Baumann, 2002; Spinelli et al., 2017). The preference for the Cantonese contour Tone 25 may thus be interpreted as an indication that the 18-month-old German infants have started to learn about these pragmatic functions of rising contours. We will discuss this point in more detail in the general discussion.

## Experiment 3: testing 6- and 9-month-olds using a habituation procedure

### Methods

#### Participants

Thirty monolingual German-learning infants participated in this experiment: 15 6-month-old (*M*_*age*_ = 182 days, *range* = 168–195 days; 8 girls) and 15 9-month-old (*M*_*age*_ = 207 days, *range* = 255–289 days; 7 girls) infants. An additional 12 infants were tested but excluded from the analysis for the following reasons: crying (*n* = 3), failure to reach the habituation criterion (*n* = 7), listening times <500 ms for at least one of the four test trials (*n* = 1), and fussiness (*n* = 1). Infants from Experiment 3 did not participate in the previous Experiment 2. All infants were born full-term and according to parental report none of the infants suffered from any repeated or acute ear infections. None of the infants showed indications of atypical development or had experience with a tone language. This study was carried out in accordance with the recommendations of the Ethics Committees of the University of Potsdam with written informed consent from all parents in accordance with the Declaration of Helsinki.

#### Stimuli

The tone contrast for this experiment was identical to the contrast in Experiment 2. For habituation and test phases, the same four tokens as used in Experiment 2 were re-arranged into new sound files. Since we had four tokens of each tone, we decided to use all tokens in the habituation and test phases in order to allow more acoustic variation within each phase. Stimuli were separated by an interstimulus interval of 1 s, resulting in a speech string of 40 s. During the experimental trials, a black and white checkerboard was displayed on a screen (e.g., Horowitz, 1974; Stager and Werker, 1997). Between trials, infants saw a silent bouncing ball to redirect their attention to the screen.

#### Procedure

Infants sat on the caretaker's lap, facing a monitor at a distance of ~ 1.2 meters in a silent room. A camera positioned above the presentation screen monitored infants' looking behavior. The stimulus presentation and infants' looking behavior was coded online using Habit 2 (Version 2.1.25, Oakes et al., 2015). All acoustic stimuli were presented with an intensity of 65 dB over loudspeakers, which were placed behind the screen. One trial consisted of a 40 s speech string. Trials started as soon as the infant fixated the screen and the experimenter pressed a key. The length of each trial was controlled by the infant's behavior: the trials ended when infants either looked away for more than 2 s, or the maximum trial duration was reached.

The experiment consisted of three phases: habituation, test, and post-test phase. The maximum number of trials within the habituation phase was 18 trials. The habituation criterion was reached when infants' mean listening time across three consecutive trials decreased to 50% of the mean listening time of the first three trials. Infants who did not reach the criterion were excluded from the analysis. All infants were habituated with Tone 25. The test phase started immediately after infants reached the habituation criterion or after the maximum number of trials was presented. In the test phase two trials with the novel (Tone 33) and two trials with the habituated (Tone 25) tone, each with a maximum duration of 40 s, were presented. The presentation order of the two novel and habituated tone trials was counterbalanced across infants. Half of the infants started the test phase with a trial containing the novel tone and the other half with a trial containing the habituated tone. A post-test phase followed directly after the test phase. During the post-test phase, a completely novel auditory stimulus was presented to verify the infants' attention to the task. The post-test trial differed segmentally from the tone stimuli. In total, 50% of the participants (randomly selected) were re-coded (frame by frame, 25 fps) by a second coder using the specialized software ELAN (Wittenburg et al., 2006). The inter-coder reliability was *r* = 0.98, *p* < 0.001.

### Results

**Figure 4:**
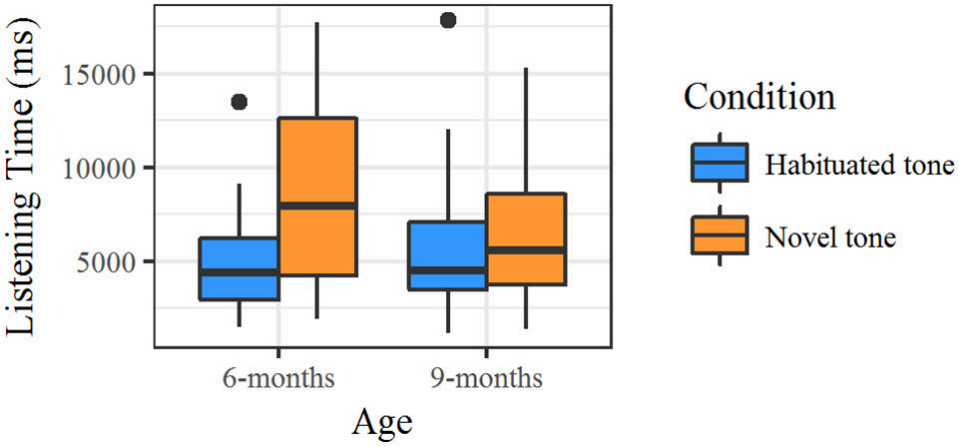
Results from the habituation experiment divided by age group. Mean listening times to the novel tone were significantly longer compared to those to the habituated tone.

The averaged listening times for the novel and the habituated stimuli served as dependent variable. Mean listening times to the different trial types for the two age groups are displayed in Figure 4. Discrimination is indicated by a longer listening time for either the novel or the habituated tone. On average, infants needed about 6.08 trials (*SD* = 4.1) to reach the habituation criterion. Both age groups accumulated a comparable amount of listening time to the stimuli during habituation (91.95 s at 6 months, and 91.55 s at 9 months).

Again, all listening times were logarithmically transformed to fulfill the assumption of normal distribution of the residuals. The statistical analysis was performed with R (R Core Team, 2017) by using linear mixed models with the lmer function from the package lme4 (Bates et al., 2015). Again, we compared different models in order to test the best model fit using the anova function. The results from a Chi-square test as well as the lowest AIC revealed best fit for a model including the interaction of Age Group (6- and 9-months) and Condition (novel and habituated tone) as fixed factor and subject as random factor. In contrast to Experiment 2, trial number did not lead to a better model fit and was therefore excluded from further analysis. The missing effect of trial number was probably due to the smaller number of test trials. For details on the statistical analysis, see Table 6.

**Table 6:** Results from the model comparison of the habituation paradigm.

**Model**	**Df**	**AIC**	**BIC**	**logLik**	**Deviance**	**ChisqChi**	**Df**	**Pr(>Chisq)**
~Condition + (1|subject)	5	345.11	360.13	−167.55	335.11			
~Condition^*^Age + (1|subject)	8	341.99	366.02	−163.00	325.99	9.12	3	0.028^*^
~Condition^*^Age + (1|subject) + (1| trial_number)	9	343.99	371.03	−163.00	325.99	0	1	1

Results from the model comparison of the habituation paradigm. The comparison is organized hierarchically. The first model was compared to the second model – which fit better to the data. The second model was then compared to the third. The second model fit best to the data and included Age and Condition as fixed effects and subject as random effects. In contrast to Experiment 2, trial number did not lead to a better model fit and was therefore excluded from further analysis. Note that habituation type was not included in the models because all infants were habituated with the same tone (Tone 25) (

* *indicates p < 0.05)*.

Since the interaction of Condition × Age Group was found to be significant, we performed separate analyses for each age group. Detailed statistical information can be found in Table 7. All comparisons were also calculated with the lmer function with Condition as fixed factor and subject as random factor. The 6-month-olds showed significantly longer listening times to the novel tone (*M* = 8.52 s, *SD* = 5.24 s) compared to the habituated tone (*M* = 5.11 s, *SD* = 4.30 s). In contrast, the 9-month-olds' listening times to the novel tone (*M* = 6.31 s, *SD* = 5.15 s) were not significantly different from those to the habituated tone (*M* = 5.98 s, *SD* = 4.12 s). Effect sizes (Cohen's *d*) were calculated for the 6-month-olds, *d* = −0.435, and for the 9-month-olds, *d* = 0.048.

**Table 7:** Detailed results from the statistical analysis of the habituation experiment for each age group.

**Fixed effects**	**Estimate β (SE)**	**df**	**t-value**	**Pr(>|t|)**
**6-MONTH-OLDS**				
Intercept (Habituated_tone)	8.328 (0.162)	24.95	51.437	<0.001^***^
Novel_tone	0.337 (0.158)	45.0	2.136	0.038^*^
**9-MONTH-OLDS**				
Intercept (Habituated_tone)	8.386 (0.17576)	23.92	47.715	<0.001^***^
Novel_tone	0.040 (0.164)	45	0.243	0.81

Detailed results from the statistical analysis of the habituation experiment for each age group. The estimates represent the log-transformed listening times. Results indicated that the 6-month-olds discriminate between Tone 25 and Tone 33, but the 9-month-olds do not. All separate models included Condition as fixed effect and subject as random effect (

* indicates p < 0.05,

*** *indicates p < 0.001)*.

### Discussion

Our results from the habituation experiment clearly show an age-related decline in perceptual sensitivity for the contrast of Cantonese high-rising and mid-level lexical tones. While the 6-month-olds succeed in discriminating the tones, the 9-month-olds did not show any evidence of discrimination. The decline in perceptual sensitivity between 6 and 9 months is in line with previous studies on lexical tone perception in infants (Mattock and Burnham, 2006; Mattock et al., 2008; Liu and Kager, 2014). These findings support the idea of perceptual reorganization for lexical tones between the ages of 6 and 9 months (Mattock and Burnham, 2006; Mattock et al., 2008; Yeung et al., 2013) and extend this observation to German-learning infants.

## General discussion

The studies presented here pursued two main goals. The first one was to investigate whether further evidence can be obtained for a U-shaped development in the discrimination of non-native tone contrasts that is characterized by an initial decline and a later re-increase of perceptual sensitivity. The second goal was to investigate whether a procedure that involves habituation in the exposure phase of the experiment provides clearer evidence of infants' discrimination of lexical tones than a procedure that uses familiarization during the exposure phase of the experiment.

Summarizing the results across the three experiments, our overall findings suggest a U-shaped developmental pattern for tone discrimination in speakers and learners of German. First, German adults are able to discriminate the Cantonese high-rising vs. mid-level tones although their performance was below that of native Cantonese speakers. Second, we found a decline in the ability to discriminate these tones between the ages of 6 and 9 months: while 6-month-olds showed a clear dishabituation and thus discrimination effect in our last experiment, the results from the 9-month-olds did not indicate any discrimination of the tones across the two experiments. Third, evidence for a decline between the ages of 6 and 9 months was only obtained after habituation, but not after familiarization. We will first discuss the implications of our findings for the understanding of perceptual reorganization in infants and then consider methodological implications.

## Understanding developmental trajectories for tone discrimination

Overall, the results from our study suggest a developmental trajectory in the tone discrimination of German-learning infants that is identical to what Liu and Kager (2014) found for Dutch-learning infants: good discrimination at 6 and 18 months of age, but not at 9 months. Our study extends the findings from Liu and Kager (2014), who used the Mandarin high-level and high-falling tones, to a different tone contrast from another language and to learners of a different L1. This is an important finding as it shows that the U-shaped developmental pattern that was reported for the first time by Liu and Kager (2014) can be replicated and does indeed generalize to a new tone type. In addition, our study revealed that the tone contrast that was used in our infant study can also be discriminated by adult speakers of German, but on a significantly lower level than by native speakers of Cantonese. Contrastingly, for other tone contrasts tested in Experiment 1, discrimination reached native-like performance in adult speakers of German. This suggests that the adult discrimination of Tone 25 and Tone 33 is not only based on the acoustic saliency of the phonetic contrast. This in turn suggests that the U-shaped developmental pattern for this tone contrast is based on perceptual reorganization influenced by the acquisition of phonological properties of the native language and is not only due to a change in the acoustic sensitivity to pitch information.

As already discussed in previous studies (Liu and Kager, 2014; Ramachers et al., 2017; Shi et al., 2017), we assume that the intonation system of the native language and the relation of the tested non-native tone contrast to this system is crucial. Changes in pitch contours are not a unique characteristic of tone languages, as they are also relevant for the intonation of languages like German. In intonation languages, pitch movements have post-lexical functions indicating prosodic (and syntactic) phrasing and pragmatic functions, such that infants growing up with a non-tone language are not fully naïve to pitch variations. In German, rising pitch contours with a nuclear pitch accent (L^*^H) are related to sentence internal boundaries of prosodic phrases and to Yes-No Questions (Grice and Baumann, 2002; Gussenhoven, 2004; Petrone et al., 2017). Since questions are frequently used in communication with infants and toddlers to catch their attention (Spinelli et al., 2017), and even infants and toddlers show discrimination of question over declarative intonation contours (Geffen and Mintz, 2011; Soderstrom et al., 2011), our finding that German toddlers discriminate high-rising from mid-level tones at 18 months of age lines up with findings from other studies that assume that the native language intonation system has an impact on lexical tone perception in speakers of non-tone languages. Their growing knowledge of German intonation and its relation to the syntactic and pragmatic system may have sharpened, or re-sharpened, their processing of the tonal information in the Cantonese stimuli. However, 5-month-old English-learning infants can discriminate between statements and questions marked by their different prosodic contours (flat vs. rising contour: Geffen and Mintz, 2011; Soderstrom et al., 2011) and German 8-month-olds can detect phrase boundaries that are marked by pitch changes in combination with final lengthening (Wellmann et al., 2012). Given these results, the question arises why a decline in perceptual sensitivity to pitch as marking lexical tone is observed in learners of non-tone languages.

If the assumption that growing knowledge about the language-specific intonation system affects tone discrimination is correct, then the discrimination abilities of 6-month-olds and that of 18-month-olds probably do not rely on the same mechanisms. Discrimination of non-native contrasts in young infants has typically been attributed to extremely sensitive acoustic perception in early development (Aslin et al., 1998), which allows the discrimination of all kinds of minimal sound contrasts. Perceptual reorganization then maintains or sharpens the discrimination of contrasts that are relevant in the linguistic system of the native language, but leads to a decline in the discrimination of sound contrasts that are not relevant in the linguistic system. Thus, we assume that the younger infants still process tone stimuli in a more acoustic manner, and while an infant's native language is expected to influence these results (cf. Yeung et al.'s, 2013 findings of language-specific differences in preferences for pitch contours across languages at 4 months of age), there should not be any decline in the ability to perceive differences in contours until a point in the development when infants must learn the linguistic functions of either tonal or intonational contrasts.

The results from the experiment using the habituation procedure with 6- and 9-month-old German infants, along with prior work illustrating the classic pattern of perceptual reorganization, suggest that 9 months of age is perhaps a critical age of interest (Mattock and Burnham, 2006; Mattock et al., 2008; Yeung et al., 2013; Liu and Kager, 2014). Because our (null) results for 9-month-olds were obtained across both experimental paradigms, we do not consider them to be a reflection of methodological issues. We propose that this decrease in tone discrimination around 9 months is an indication of a milestone in infants' linguistic development, when infants begin to reorganize their perceptual systems to understand how pitch is functionally used in their target language, with an emphasis on word-level meanings. For infants learning German, within-syllable pitch information is not lexically informative, and so like other 9-month-old learners of non-tonal languages, they may start to ignore pitch cues from this age.

A study by Hay et al. (2015) provides data that is related to this general idea. They found that 14-month-old English-learning infants can still use a Mandarin rising and falling tone contrast in word learning by mapping novel objects to labels that differ only in pitch contours. However, 17- and 19-month-olds tested with the same procedure did not respond to this labeling violation (for similar results with English-learning 2-year-olds, see Quam and Swingley, 2010). Testing the 19-month-olds on pure discrimination of the tones using a habituation task further revealed that these older infants could nevertheless discriminate the target tones. Hay et al. (2015) discuss this change across ages as an indication that infants get increasingly more specific about the sound contrasts that they consider to be lexically contrastive. Therefore, the older toddlers do not attend to tone contrasts in a word learning scenario, although they can discriminate them in other contexts. Infants and toddlers in our study were younger, but it may still be the case that their performance reflects shifts in attention related to lexical development. As Bergelson and Swingley (2012) have shown, infants from 6 to 9 months of age may already be strongly focused on word learning, and may be particularly attuned to sound contrasts that are lexically contrastive in their language (i.e., German), while largely ignoring sound contrasts that are not. In intonation languages, attention to tonal information may then potentially increase again when children start to detect semantic or pragmatic functions of the intonational patterns in their language which could explain why at 18-months German and Dutch infants again showed discrimination of the lexical tones. Further research would be necessary to test this hypothesis.

Future research must explore these ideas further, as lexical development might not be the only factor explaining the dip in discrimination abilities. Other factors, like salience of the contrast, might interact with the lexical development: for example, previous tone discrimination studies have not shown a perceptual decline at 9 months for certain tone contrasts (e.g., Liu and Kager, 2014, 2017; Ramachers et al., 2017; Shi et al., 2017; Tsao, 2017). Relatedly, a perceptual shift has also been reported in the visual domain around the same age, suggesting parallel development across perceptual domains. Data from Lewkowicz and Hansen-Tift (2012) have also shown a U-shaped function in visual scanning, such that infants around 8 to 9 months of age look at the mouth, whereas 4- and 12-month-olds look at the eyes. This shift may be symptomatic of a general increase in attention to certain units (segmental relative to suprasegmental information). Much remains unclear about why infants from 8 to 10 months of age show a specific developmental pattern with respect to tone perception.

## Methodological comparisons

The difference in the 6-month-olds' results between the familiarization and the habituation experiment line up with previous research, since most other studies have shown lexical tone discrimination with habituation procedures (Liu and Kager, 2014, 2017; Chen and Kager, 2016; Ramachers et al., 2017; Shi et al., 2017; Tsao, 2017), whereas a decline in perceptual sensitivity has mostly been found with studies using a familiarization procedure (Mattock and Burnham, 2006; Mattock et al., 2008; Yeung et al., 2014). Similar to the findings from Cristia et al. (2016), our results show that the habituation procedure generates larger effect sizes at the group level. Both habituation and familiarization procedures are based on the customization of the participants to one type of stimulus and then measuring differences in the response to the old vs. a new stimulus. As stated in the introduction, we assume that habituation procedures are more adapted to individual variation by only stopping the initial exposure phase when the behavior of the infant indicates a specific level of customization. In contrast, familiarization-based procedures use a fixed amount of time or number of presentations and do not take individual differences in processing the stimuli into account. A comparison of the exposure time in our two experiments shows large differences: recall that the familiarization in Experiment 2 was fixed to 30 s of exposure to one of the tones. However, in the habituation experiment, infants needed about six presentation trials and accumulated an overall listening time to the tones of about 90 s before they reached the criterion, suggesting that they had more exposure to the crucial stimulus then the infants in Experiment 2. This difference may explain why the 6-month-olds discriminate the two tones after habituation, but not after familiarization: the amount of exposure may not have been sufficient for this age group to encode the stimulus in a way that allowed for its discrimination from another stimulus during the test phase. This also suggests that 6-month-olds may show discrimination after a longer familiarization [for effects of familiarization duration on infants' discrimination performance, see Bijeljac-Babic et al. (2012)]. The effect of trial number observed for Experiment 2 corroborates these considerations. Across the test phase, the listening times in 6-month-olds changed: while there was no evidence of discrimination in the first four trials, infants showed significantly different listening times to the two tones in the last trials^3^. This change over the experiment did not hold for the 9-month-olds^4^, which underlines that the discrimination performance by the 9-month-olds was not affected by the methodological modulation but that the effects of perceptual reorganization are rather robust in this age group.

However, it can also be the case that other reasons might explain the different findings in our two experiments: for example, the higher number of different trial types in the SAPP may have made infants' responses less sensitive across the conditions. The SAPP as used in our experiment and in the study by Yeung et al. (2013) included three trial types (one non-alternating containing the familiarized tone, one non-alternating containing the novel tone, and one alternating), whereas the studies that used habituation during the initial exposure phase only presented two different trial types, as we did in our Experiment 3 (habituated tone and novel tone: Chen and Kager, 2016; Chen et al., 2017; Shi et al., 2017; or habituated tone and alternating: Ramachers et al., 2017), or only one trial type (the novel tone: Liu and Kager, 2014, 2017) during the test phase. Our two experiments with the infants also differed in another aspect of the experimental procedure. In Experiment 2, the duration of a head-turn to the presentation side of the acoustic stimulus was measured, while in Experiment 3 we measured visual fixation on a central monitor. We consider it unlikely that this methodological difference was responsible for the differential results across the two experiments, since listening times were the dependent variable in both cases. Moreover, head-turning vs. visual fixation was not considered as a highly relevant factor in modulating test-retest reliability data in the analysis by Cristia et al. (2016).

However, the difference in the results of our experiments across the two testing conditions underlines the importance of the methodological decisions made for experiments with infants. To make research undertaken by different labs more comparable, a higher standardization of the methods used for specific research questions is desirable. We agree with Cristia et al. (2016) that this is specifically important for infant research as it is slow and costly, and therefore needs the close collaboration of researchers across institutions and languages.

## Conclusions

Taken together, our findings suggest an age-related decline in the discrimination of lexical tones between 6 and 9 months with an additional perceptual recovery at the age of 18 months in German-learning infants. The perceptual recovery in toddlers might be driven by their acquisition of the native intonation and pragmatic system, whereas the discrimination at 6 months of age may be attributed to universal listening abilities. The decline in the ability to discriminate a non-native contrast was only evident when using habituation, but not when using familiarization, suggesting that methodological aspects are important to consider in the interpretation of findings from infant studies.

## Author contributions

AG contributed to the design of the work, acquisition and analysis of the data, and drafting of the work. AK contributed to the design of the work, and revising of the manuscript. GS contributed to the design of the work, and revising of the manuscript. HY contributed to the design and stimuli construction, as well as to the revising of the manuscript. BH contributed to the design of the work, interpretation of the data, and drafting and revising of the manuscript. All authors gave final approval of the version to be submitted. All authors approved the final version and agreed to be accountable for all aspects of the work in ensuring that questions related to the accuracy or integrity of any part of the work are appropriately investigated and resolved.

### Conflict of interest statement

The authors declare that the research was conducted in the absence of any commercial or financial relationships that could be construed as a potential conflict of interest.

## Footnotes

^1^Limburgian is a dialect of Standard Dutch that uses word-level pitch for marking lexical and grammatical differences.

^2^This tone contrast was also used in the study by Yeung et al. (2013) that tested English-learning infants. Given the prosodic similarity between English and German, we expected this tone contrast to generate similar effects in German-learning infants.

^3^First four trials: Alternating vs. Non-Alternating Tone 25 (β = 0.112, *SE* = 0.148, *t* = 0.760, *p* = 0.45), Alternating vs. Non-Alternating Tone 33 (β = 0.034, *SE* = 0.148, *t* = 0.233, *p* = 0.82). Last four trials: Alternating vs. Non-Alternating Tone 25 (β = 0.226, *SE* = 0.111, *t* = 2.017, *p* = 0.04), Alternating vs. Non-Alternating Tone 33 (β = 0.121, *SE* = 0.111, *t* = 1.084, *p* = 0.28).

^4^First four trials: Alternating vs. Non-Alternating Tone 25 (β = −0.020, *SE* = 0.148, *t* = −0.137, *p* = 0.89), Alternating vs. Non-Alternating Tone 33 (β = −0.183, *SE* = 0.148, *t* = −1.242, *p* = 0.22). Last four trials: Alternating vs. Non-Alternating Tone 25 (β = 8.5e-03, *SE* = 1.1e-01, *t* = 0.073, *p* = 0.94), Alternating vs. Non-Alternating Tone 33 (β = 9.8e-05, *SE* = 1.1e-01, *t* = 0.001, *p* = 0.99).
